# Evolution of Influenza A(H3N2) Viruses in Bhutan for Two Consecutive Years, 2022 and 2023

**DOI:** 10.1111/irv.70028

**Published:** 2024-10-23

**Authors:** Tshering Dorji, Kunzang Dorji, Sonam Gyeltshen

**Affiliations:** ^1^ National Influenza Centre (NIC), Royal Centre for Disease Control, Ministry of Health Royal Government of Bhutan Thimphu Bhutan

**Keywords:** Bhutan, H3N2 subtype, influenza A virus, molecular evolution, mutation

## Abstract

**Background:**

Influenza A viruses pose a significant public health threat globally and are characterized by rapid evolution of the hemagglutinin (HA) gene causing seasonal epidemics. The aim of this study was to investigate the evolutionary dynamics of A(H3N2) circulating in Bhutan during 2022 and 2023.

**Methods:**

We analysed 166 whole‐genome sequences of influenza A(H3N2) from Bhutan, obtained from the GISAID database. We employed a Bayesian Markov Chain Monte Carlo (MCMC) framework, with a curated global dataset of HA sequences from regions with significant migration links to Bhutan. Phylogenetic, temporal, and phylogeographic analyses were conducted to elucidate the evolutionary dynamics and spatial dissemination of the viruses.

**Results:**

Our phylogenetic analysis identified the circulation of influenza A(H3N2) Clade 3C.2a1b.2a.2 in Bhutan during 2022 and 2023, with viruses further classified into three subclades: 2a.3 (39/166), 2a.3a.1 (58/166) and 2a.3b (69/166). The TMRCA estimates suggest that these viral lineages originated approximately 1.93 years prior to their detection. Phylogeographic analysis indicates introductions from the United States in 2022 and Australia in 2023. The mean evolutionary rate across all gene segments was calculated to be 4.42 × 10^−3^ substitutions per site per year (95% HPD: 3.19 × 10^−3^ to 5.84 × 10^−3^), with evidence of purifying selection and limited genetic diversity. Furthermore, reassortment events were rare, with an estimated rate of 0.045 events per lineage per year.

**Conclusion:**

Our findings show that primary forces shaping the local evolution of the influenza A(H3N2) in Bhutan are largely stochastic, with only sporadic instances of adaptive change, and thus underscore the importance of continuous surveillance to mitigate the impact of evolving strains.

## 1 Introduction

Human influenza viruses are a significant cause of morbidity and mortality worldwide. On average, they account for infections in 5–15% of the global population and cause between 290,000 and 650,000 respiratory deaths annually [1]. Incidence of influenza episodes and influenza‐associated ALRI is found to be highest in children after the first year of life in children aged 0–4 years with Influenza A (particularly H3N2 subtype), resulting in higher morbidity and mortality than does influenza B [2]. In Bhutan, an estimated 50 per 100,000 persons (95% CI: 45–55) in 2015 and 118 per 100,000 persons (95% CI: 110–127) in 2016 were hospitalized due to influenza‐associated respiratory hospitalizations with the highest rates among children <5 years [3].

Influenza viruses are an enveloped virus belonging to the Orthomyxoviridae and are of four genera that infect vertebrates including influenza virus A, B, C and D. Influenza A viruses (IAVs) are important veterinary and human health pathogens and are most virulent virus causing respiratory disease [4]. The genome of the influenza A virus (IAV) is composed of eight single‐stranded viral RNA segments. These segments are packaged together into a single virus particle and are contained in separate viral ribonucleoprotein (vRNP) complexes, in which the 5′ and 3′ termini are bound by a viral RNA‐dependent RNA polymerase (RdRp), and the rest of the RNA is bound by oligomeric viral nucleoprotein (NP), forming a double‐helical rod‐like structure [5, 6]. Segments 1–8 are named according to the encoded protein:polymerase basic protein 2 (PB2), polymerase basic protein 1 (PB1), polymerase acidic protein (PA), hemagglutinin (HA), nucleoprotein (NP), neuraminidase (NA), matrix protein (M) and nonstructural protein (NS). The surface HA and NA glycoproteins of influenza A viruses determine their reactivity and classification into 18 HA subtypes (H1–H8) and 11 NA subtypes (N1–N11), resulting in 144 possible combinations. Influenza B viruses, however, do not have any subtypes. The influenza A viruses currently circulating in humans are subtypes A(H1N1) pdm09 and A(H3N2) [7]. IAV undergoes rapid evolution through both mutation and reassortment mechanisms. Within each virus subtype, the gradual accumulation of nucleotide and amino acid substitutions in the HA and NA surface glycoproteins periodically leads to the emergence of new antigenic variants, a phenomenon known as antigenic drift. A(H3N2) viruses exhibit a higher frequency of generating new antigenic variants, occurring every 3–5 years, compared to the less frequent appearance of new variants in A(H1N1) and influenza B viruses (at intervals of 3–8 years) [8, 9, 10]. Moreover, the process of genetic exchange, facilitated by reassortment of segmented genomes, frequently imparts novel genetic characteristics to IAVs. Such genetic alterations have the potential to influence the transmissibility and pathogenicity of these viruses [11].

Since its emergence in 1968, the influenza A(H3N2) virus has shown rapid evolutionary dynamics, contributing to recurrent severe epidemics, diminished vaccine efficacy and frequent antigenic drift. In this study, we investigate the evolutionary history of the A(H3N2) viruses that circulated in Bhutan in 2022 and 2023. By conducting a thorough analysis of Bhutanese A(H3N2) genomes, we aim to elucidate the seasonal evolutionary patterns and pinpoint key mutations. Monitoring viral antigenicity through detection of important genetic changes is essential for ensuring that vaccine strains remain well matched, thereby enhancing influenza surveillance and prevention strategies.

## 2 Materials and Methods

### 2.1 Dataset Preparation

Bhutan's influenza surveillance programme started in November 2008 with three sentinel sites and expanded to 11 sites by 2012 to monitor Influenza‐like Illness (ILI) and Severe Acute Respiratory Infections (SARI) [12]. In 2014, the surveillance guidelines were revised to reduce the number of ILI sites to seven, while maintaining the SARI sites. Selected positive clinical samples from this national programme are sent quarterly to the US‐CDC, the WHO Collaborating Centre in Atlanta for sequencing and further characterization. For this study, we utilized Influenza A(H3N2) sequences from the years 2022 and 2023, which were downloaded from the Global Initiative on Sharing All Influenza Data (GISAID) (https://gisaid.org/). We prepared a total of three datasets for this study. The first dataset (Dataset 1) includes all HA sequences reported in Bhutan in 2022 and 2023 (*n* = 166), used for phylogenetic analysis and clade identification. We also downloaded the WHO recommended vaccine strains for the northern hemisphere (NH) (*n* = 6) and reference viruses (*n* = 24) to construct a maximum‐likelihood (ML) tree. The second dataset (Dataset 2) comprises of all eight gene segments of influenza A(H3N2) strains from Bhutan within the same period, facilitating evolutionary analysis. The third dataset (Dataset 3) consists of HA sequences of A(H3N2) from Bhutan, along with a curated global dataset of HA sequences from 2022 and 2023, facilitating the investigation of global phylogeographic analysis and the potential origins of the influenza A(H3N2) strains isolated in 2022 and 2023. Table S1 includes the EPI_SET ID and the acknowledgement for each influenza A(H3N2) sequences used in this study.

### 2.2 Phylogenetic Analysis and Clade Identification

The HA gene sequences from Dataset 1 were aligned using MUSCLE in MEGA 11 [13]. A maximum likelihood (ML) phylogenetic tree was constructed using IQ‐TREE webserver (http://iqtree.cibiv.univie.ac.at/), employing the best fit nucleotide substitution model (TVM + F + G4). The robustness of the tree was evaluated using 1000 ultrafast bootstrap replicates, with branch support indicated for bootstrap values greater than 80% on the consensus tree. Mutations in the HA gene were characterized relative to the vaccine strain A/Darwin/6/2021 (EPI_ISL_3534319), used for the 2022–2023 and 2023–2024 NH influenza seasons, using Nextclade (https://clades.nextstrain.org/).

### 2.3 Temporal Signal and Evolutionary Rate Analysis

The ‘nonclock’ ML tree, derived from phylogenetic analysis, served as the input for TempEst v1.5.3 [14] to test the temporal signal necessary for time‐scaled analysis. This was achieved by regressing root‐to‐tip genetic distances against the dates of sample collection. Bayesian Markov Chain Monte Carlo (MCMC) method in BEAST (v1.10.4) [15] was used to estimate the rate of nucleotide substitutions per site per year, Time to the Most Recent Common Ancestor (TMRCA) and relative genetic diversity expressed as (*N*_*e*_ *t*), where (*N*_*e*_) is the effective population size and (*t*) is the generation time. We selected a strict molecular clock model with coalescent Bayesian skyline prior and the HKY + Γ4 nucleotide substitution model [16] based the best‐fitting model for nucleotide substitution determined by JModel test [17]. The MCMC chains were run for 100 million iterations, with subsampling at every 10,000 iterations. We assessed the convergence using Tracer v1.7.1 [18], and the effective sample size (ESS) of more than 200 was accepted. Uncertainty in the estimates was indicated by 95% highest posterior density (95% HPD) intervals. We summarized the posterior distribution of the trees using TreeAnnotator v1.10.4 after discarding burn‐in of 10% and visualized the maximum clade credibility (MCC) tree using FigTree v1.4.4 (http://tree.bio.ed.ac.uk/software/figtree/).

### 2.4 Reassortment and Selection Pressure Analysis

The CoalRe package in BEAST2 (v2.7.6) [19] was used to estimate the intralineage reassortment networks between H3 and N2. MCMC sampling was run for 500 million steps and performed following the tutorial (https://taming‐the‐beast.org/tutorials/Reassortment‐Tutorial). The embed segment tree was drawn using icytree (https://icytree.org/) to depict the reassortment network between HA and NA. The potential sites under selection were tested on the Datamonkey server (https://www.datamonkey.org/) [20] by calculating the ratio of nonsynonymous (dN) and synonymous (dS) nucleotide substitutions per site (dN/dS). Four site‐level detection methods, namely, Fixed Effects Likelihood (FEL), Single‐Likelihood Ancestor Counting (SLAC), Fast Unconstrained Bayesian AppRoximation (FUBAR) and Mixed Effects Model of Evolution (MEME), were used to assess positive and negative selection codons. We considered sites with a *p* value less than 0.1 as positively selected.

### 2.5 Global Phylogeographic Analysis

To understand the transmission dynamics and elucidate the possible geographical origins of the Influenza A(H3N2) viruses in Bhutan, we curated a dataset of global HA sequences from GISAID collected in 2022 and 2023 (Dataset 3). We subsampled sequences from key regions including North America (United States, *n* = 92), Oceania (Australia, *n* = 152), East and Southeast Asia (Singapore, *n* = 44; Bangladesh, *n* = 26; India, *n* = 55; Hong Kong, *n* = 7; Thailand, *n* = 24) and Europe (United Kingdom, *n* = 8). Key regions were selected based on international travel patterns into Bhutan from the Statistical Yearbook of Bhutan 2023 [21] to optimize computational efficiency. We filtered the sequences for phylogeographic analysis to include only those from clades corresponding to the Bhutanese strains, identified through Nextstrain. A Bayesian phylogenetic analysis was then conducted with BEAST (v1.10.4) assuming a strict molecular clock. We employed a discrete trait substitution model with a symmetric substitution model and applied Bayesian Stochastic Search Variable Selection (BSSVS) to infer the social network [22]. The phylogeographic inferences of influenza virus using HA segments were analysed and visualized with the spatial phylogenetic reconstruction of evolutionary dynamics using SPREAD v1.0.7 [23]. A keyhole markup language (KML) file was generated and visualized via Google Earth (https://www.google.com/earth). The final rate matrix log files were used to calculate BF values for significant diffusion rates between discrete locations. We considered a predictor with a BF > 3 as having significant support.

### 2.6 Ethical Approval

The work described here is a retrospective study performed entirely on data obtained from a publicly available database, GISAID. No additional demographic or clinical information was used in this study. Therefore, informed consent or ethical clearance was not applicable.

## 3 Results

### 3.1 Phylogenetic Analysis and Clade Identification

**FIGURE 1:**
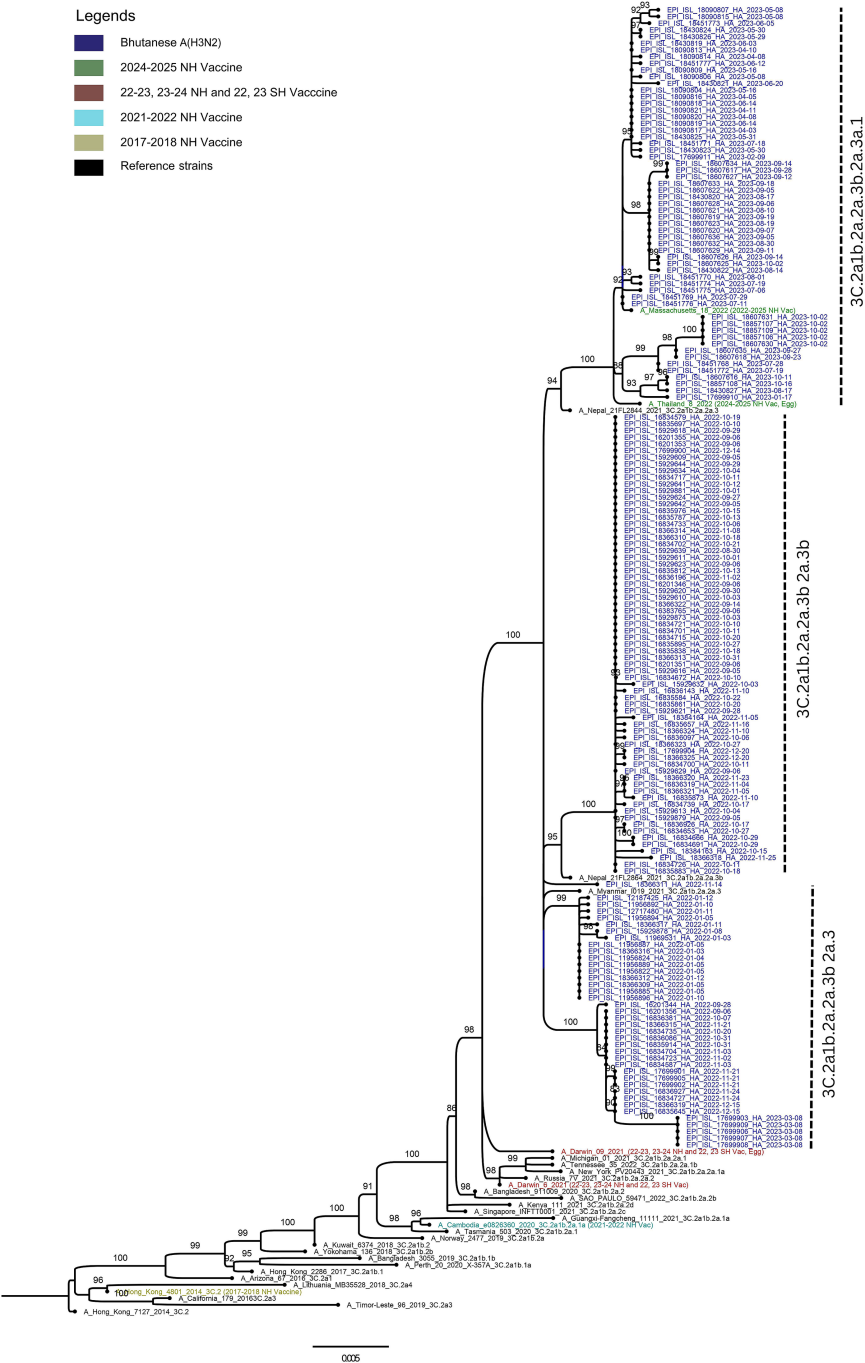
Maximum‐likelihood (ML) phylogenetic tree of the hemagglutinin (HA) sequences of A(H3N2) strains from Bhutan during the period of study. The WHO recommended vaccine A(H3N2) strains of the northern hemisphere and reference clades from GISAID were included and annotated in the trees. The Bhutanese strains that belonged to 3C.2a1b.2a.2 are annotated in blue, and reference clades were shown in black. The phylogenetic tree was inferred by the maximum‐likelihood method using 1000 bootstrap replicates implemented in IQ‐TREE webserver. Branch values of >80% are indicated at the nodes.

**FIGURE 2:**
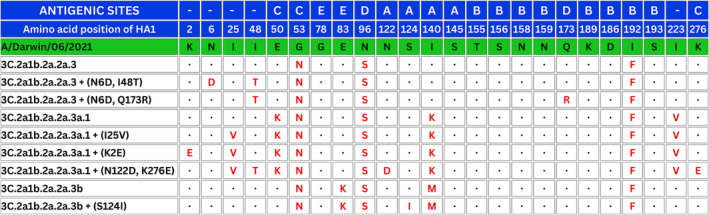
Sequence alignment showing mutations in selected positions of HA sequences of Bhutanese influenza A(H3N2) strains. In the sequence alignment, mutations in specific positions of HA sequences from Bhutanese influenza A(H3N2) strains are highlighted. Dots indicate amino acids similar to those in the A/Darwin/06/2021 vaccine strain, whereas residue changes are highlighted in bold red. The amino acid positions are labelled with the corresponding antigenic sites.

The phylogenetic analysis of the HA gene of Bhutanese influenza A(H3N2) viruses collected in 2022–2023 and 2023–2024 influenza seasons demonstrated that viruses primarily belonged to Clade 3C.2a1b.2a.2. They were further categorized into three major subclades: 2a.3 (39/166), 2a.3a.1 (58/166) and 2a.3b (69/166), as illustrated in Figure 1. When compared to the WHO recommended vaccine strain A/Darwin/6/2021 for the NH influenza seasons of 2022–2023 and 2023–2024, our genomes from Subclade 2a1b.2a.2a.3 exhibited the following amino acid substitutions: G53N, N96S‐ADD‐GLY, I192F, N378S and D513E. Similarly, Subclade 2a.3a.1 displayed mutations E50K, G53N, N96S‐ADD‐GLY, I140K, I192F, I223V and N378S, whereas Subclade 2a.3b was characterized by mutations D7N‐ADD‐GLY, D32E, G53N, N96S‐ADD‐GLY, I140M, I192F and N378S. The major mutations in the Bhutanese influenza A(H3N2) are shown in Figure 2.

### 3.2 Temporal Signal and Evolutionary Analysis

The analysis of the nonclock ML tree obtained using IQ‐TREE showed high correlation (*R* ^2^ = 0.952) between time and root‐to‐tip genetic distance (Figure S1), thus suggesting a sufficient molecular clock signals for Bayesian analysis. We analysed all available segments of influenza A(H3N2) sequenced from Bhutan in 2022 and 2023 to determine evolutionary and mutation patterns. The time to the most recent common ancestor (TMRCA) for the Bhutanese A(H3N2) strains sampled in 2022 and 2023 is estimated to be 1.93 years with 95% HPD interval of 1.81 to 2.11 years. Nucleotide substitutions in the whole genome of Bhutanese A(H3N2) viruses evolved at mean rates of 4.42 × 10^−3^ mutations per site per year (95% HPD, 3.19 × 10^−3^ to 5.84 × 10^−3^) across all eight gene segments. The mean rates of nucleotide substitution of the individual segments varied with the highest rates observed in the major glycoproteins of NS (5.96 × 10^−3^ substitutions per site per year), followed by HA segment (5.26 × 10^−3^ substitutions per site per year) and NA (5.06 × 10^−3^ substitutions per site per year). The lowest evolutionary rates were observed for the PB2 segment (2.20 × 10^−3^ substitutions per site per year). Detailed evolutionary rates and TMRCA for all segments of the genome are shown in Table 1.

**TABLE 1:** Mean rates of nucleotide substitutions, TMRCAs with credibility intervals (95%HPD), and selection pressure analysis for all segments of A(H3N2) viruses circulating in Bhutan in 2022 and 2023.

Segments (*n* = 164)	TMRCA (95% HPD)	Evolutionary rate (95% HPD)	dN/dS		Total sites	Sites under diversifying positive selection (Amino acid position)				Number of sites under purifying negative selection		
			SLAC^a^	MEME^a^		SLAC^a^	MEME^a^	FEL^a^	FUBAR^b^	SLAC^a^	FEL^a^	FUBAR^b^
HA	1.93 (1.80–2.10)	5.26 × 10^−3^ (4.07–6.67)	0.261	0.232	566	—	14, 180, 517	—	—	2 sites	33 sites	3 sites
NA	1.95 (1.80–2.10)	5.06 × 10^−3^ (4.59–6.93)	0.352	0.317	470	—	—	—	13, 329, 331	6 sites	16 sites	11 sites
MP	2.59 (1.85–3.88)	4.51 × 10^−3^ (2.76–6.42)	0.145	0.136	252	—	111	—	111	—	6 sites	5 sites
NP	3.83 (2.70–5.01)	3.12 × 10^−3^ (2.17–4.10)	0.091	0.083	498	—	—	—	—	3 sites	21 sites	37 sites
NS	2.09 (1.87–4.05)	5.96 × 10^−3^ (4.07–7.69)	0.61	0.06	230	—	—	—	22, 139	1 site	5 sites	2 sites
PA	3.34 (2.50–4.23)	3.46 × 10^−3^ (2.52–4.43)	0.119	0.109	716	—	—	—	256	2 sites	41 sites	79 sites
PB1	3.96 (2.96–4.94)	3.08 × 10^−3^ (2.34–3.80)	0.069	0.064	757	—	—	—	—	5 sites	38 sites	107 sites
PB2	4.84 (3.62–6.20)	2.20 × 10^−3^ (2.20–3.74)	0.087	0.085	759	—	109	—	—	1 site	43 sites	133 sites

Abbreviations: FEL, fixed effects likelihood; FUBAR, Fast Unconstrained Bayesian AppRoximation; MEME, mixed effects model of evolution; SLAC, single‐likelihood ancestor counting.

^a^ *p* value threshold of 0.1.

^b^ Posterior probabilities of 0.9.

**FIGURE 3:**
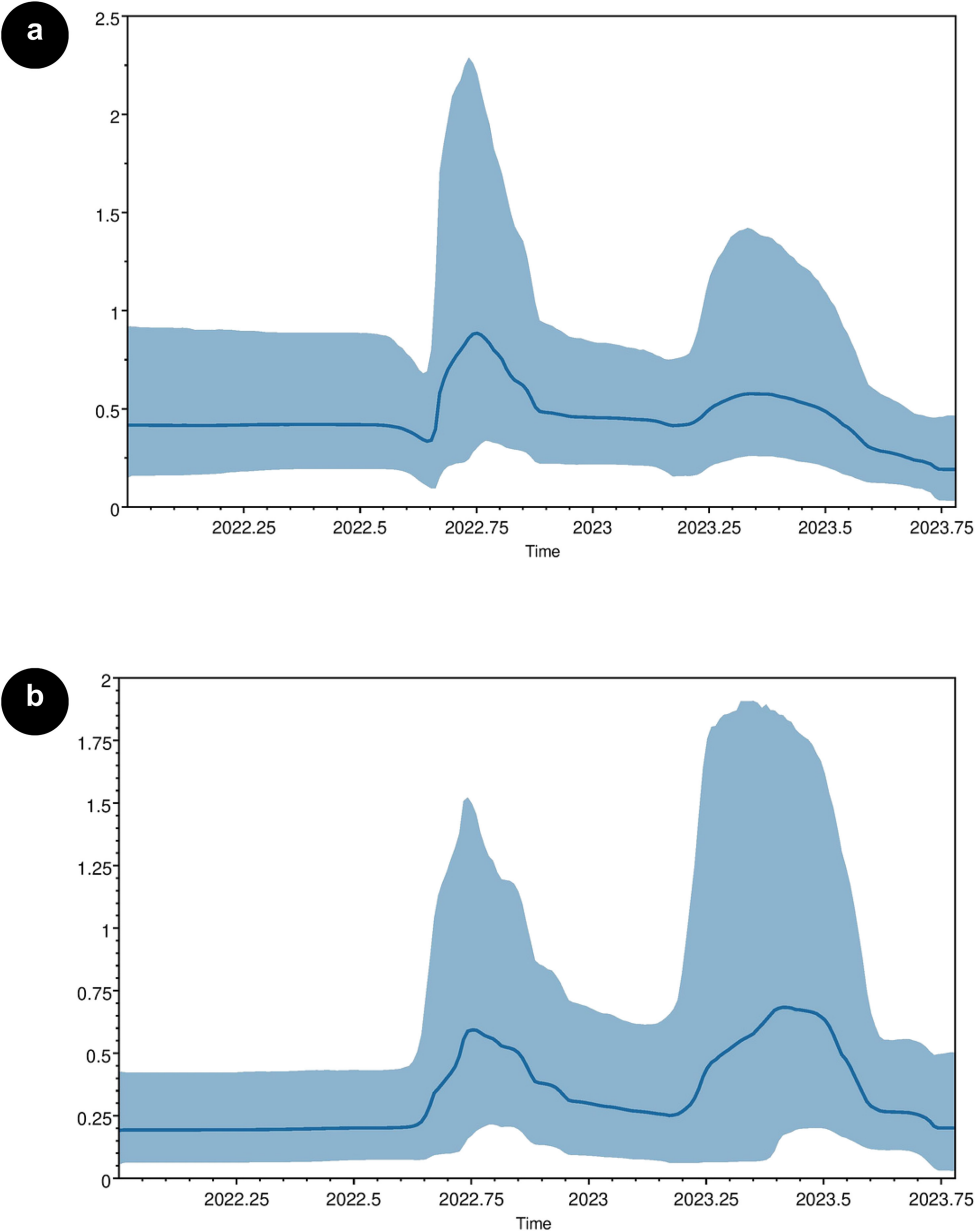
Bayesian skyline plot of HA and NA genes. This plot depicts the temporal changes in genetic diversity for HA (a) and NA (b) genes. The *x* axis shows the timeline in years, whereas the *y* axis measures relative genetic diversity, expressed as (*N*_*e*_ *t*), where (*N*_*e*_) is the effective population size and (*t*) is the generation time. The mean trend is represented by the blue line, with the shaded area indicating 95% HPD.

The Bayesian skyline plot (BSP), illustrating the changes in genetic diversity through time, showed the changing level of genetic diversity of HA and NA genes of Bhutanese A(H3N2) strains sampled in 2022 and 2023. Despite the overall low genetic diversity, two minor surges in relative diversity (*R*_*e*_) were observed in both genes, with first occuring arround August 2022, followed by another similar rise in March 2023 (Figure 3).

### 3.3 Reassortment and Selection Pressure Analysis

Global selective pressure analysis using FEL and SLAC models, applied across all eight segments at *p* ≤ 0.1, detected no evidence of positive or diversifying selection. In contrast, the MEME model identified three positively selected sites within the HA segment (14, 180 and 517) with an overall dN/dS ratio of 0.23. The SLAC model revealed a slightly higher dN/dS ratio of 0.261 for the HA segment of the Bhutanese A(H3N2) strains. Additionally, the MP and PB2 segments showed one diversifying codon each, at positions 111 and 109, respectively, using MEME model. The detailed selection test results are shown in Table 1.

The coalescent‐based analysis estimated that Bhutanese A(H3N2) strains underwent reassortment at a mean rate of 0.045 events per lineage per year (95% HPD: 0.00075–0.174). Over the study period, a single intralineage reassortment event between the H3 and N2 genes of Subclade 2a.3a.1 was identified, occurring in viral strains sampled in 2022 and 2023. This event is represented by a dotted line in the MCC tree (Figure S2).

### 3.4 Global Phylogeographic Analysis

**FIGURE 4:**
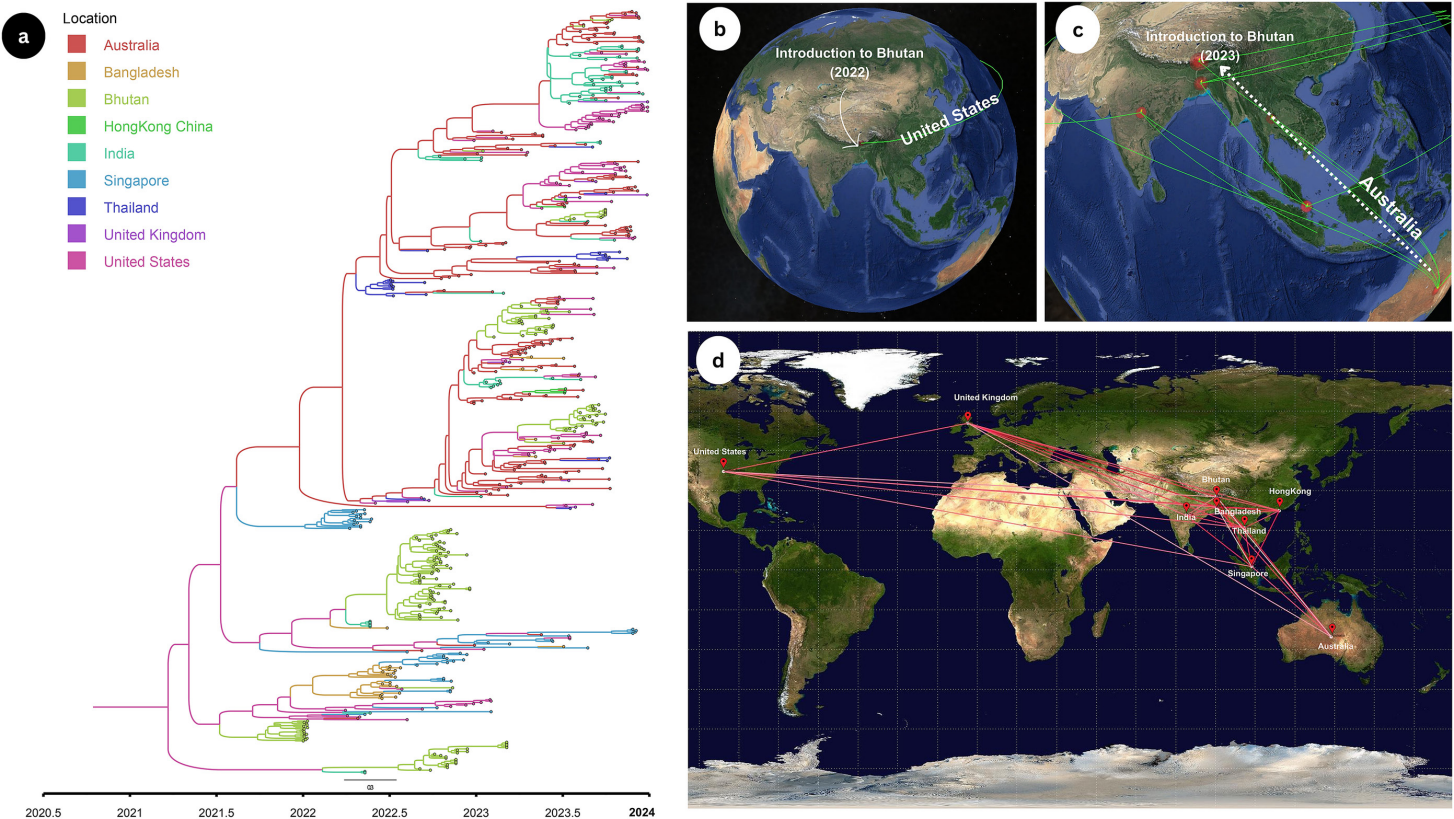
Bayesian Maximum‐clade‐credibility (MCC) tree and phylogeographic analysis of the migratory patterns of Bhutanese influenza A(H3N2) viruses. (a) Global MCC tree for influenza A(H3N2) virus in 2022 and 2023. Branches were coloured according to most probable geographic origin. (b) 2022 epidemic was introduced from the United States (c) 2023 from Australia, and (d) Bayes factor (BF) test for significant nonzero rates. Only rates supported by a BF greater than 3 are indicated. The colour of the line represents the relative strength by which the rates are supported; white suggest relatively weak and red lines depicts strong rate support.

To investigate the global migration patterns of influenza A(H3N2) in 2022 and 2023, and to identify the potential geographical origins of viruses circulating in Bhutan during this period, we performed a Bayesian phylogeographic analysis. This analysis was based on the hemagglutinin (HA) sequences from Bhutan (*n* = 166), combined with an additional curated global dataset (*n* = 408) of strains collected in 2022 and 2023. Using these data, we constructed time‐scaled phylogeographic maximum clade credibility (MCC) tree, inferring the ancestral locations of each branch by utilizing the collection dates and locations of the sequences. In 2022, Subclades 2a.3 and 2a.3b were dominant, whereas Subclade 2a.3a.1 emerged in 2023. The MCC tree indicated that the common ancestors of the A(H3N2) strains were most likely from North America, with a significant presence of strains from the United States along the phylogenetic trunk. Our analysis of the HA segment dispersal history suggests that the earliest divergence events in 2022 involved transmission from the United States to Bhutan, alongside concurrent spread to other Asian countries, including Bangladesh and India (Figure 4b–d). Figure S3 provides an animated GIF illustrating the phylogeographic spread of A(H3N2) strains into Bhutan, detailing viral transmission pathways from the United States in 2022 and Australia in 2023. Bayesian analysis showed strong transmission support between the United States and Bhutan in 2022, with a Bayes factor of 15.30. Similarly, in 2023, transmission between Australia and Bhutan was also significantly supported, with a Bayes factor of 5.0. A detailed summary of the Bayes factors and posterior probabilities for migration events of influenza A(H3N2) between Bhutan and other major regions in 2022 and 2023 is shown in Table S2.

## 4 Discussion

The IAV undergoes continuous evolution through mutations and genetic reassortment. These evolutionary processes are pivotal, as they can alter the virus's host specificity and pathogenicity [24]. Rapidly evolving pathogens like Influenza viruses exhibit a unique phenomenon where their ecological and evolutionary dynamics transpire concurrently. As a result, these rapidly mutating viral populations can develop noticeable genetic variations within mere days, enabling them to swiftly adapt to new environments [25]. To our knowledge, this is the first comprehensive whole‐genome study of influenza A(H3N2) viruses in Bhutan focusing on the evolutionary pattern of all eight gene segments of the influenza A(H3N2) virus. In this study, we analysed influenza A(H3N2) sequences collected throughout the year from January 2022 till December 2023.

Our results show that influenza A(H3N2) circulating in Bhutan primarily belonged to Clade 3C.2a1b.2a.2, specifically Subclades 2a.3a and 2a.3b in 2022 and 2a.3a.1 in 2023. These subclades have been in global circulation since 2021, consistent with global data from the 2022–2023 NH influenza season [26, 27]. When compared to the WHO‐recommended vaccine strain A/Darwin/6/2021 for the 2022–2023 and 2023–2024 NH influenza seasons, the Bhutanese strains within Subclade 2a.3 exhibited notable amino acid substitutions: G53N, N96S (with an additional glycosylation), I192F, N378S and D513E. Similarly, Subclade 2a.3a.1 showed mutations such as E50K, G53N, N96S (with glycosylation), I140K, I192F, I223V and N378S. Subclade 2a.3b, on the other hand, was characterized by D7N (with glycosylation), D32E, G53N, N96S (with glycosylation), I140M, I192F and N378S. Past reports show that the mutations responsible for major antigenic change were located exclusively in amino acid position 145 of antigenic Site A, amino acid positions 155, 156, 158, 159, 189 and 193 of antigenic Site B, and with none in Sites C, D, or E [28]. In our analysis, there were no mutations observed at these sites in the Bhutanese A(H3N2) strains, and this absence suggests a potential stability in the antigenic profile, which could have significant implications for vaccine efficacy.

We used a molecular clock approach to calculate the evolutionary rates and the TMRCA of influenza A(H3N2) viruses sequenced from Bhutan in 2022 and 2023. The TMRCA estimates of the earliest clade in 2022 suggest that it probably entered Bhutan between July 2019 and January 2020 and segregated into three subclades. Among the eight gene segments, NS showed the highest evolutionary rates followed by HA and NA genes in Bhutanese A(H3N2) viruses. Similar findings were also observed in Bangladeshi strains with NS segments showing highest substitution rates [29]. PB2, PB1, PA and NP showed similar rates and are expected to co‐evolve due to the functions of the proteins they encode. The overall rate was higher than the rates reported for A(H1N1) and influenza B viruses [30].

The analysis of selection pressure revealed that Bhutanese A(H3N2) strains are predominantly under purifying selection, with most amino acid substitutions distinguishing viral clades being neutral. Only a few sites were found to be under positive selection, suggesting that adaptive changes occur sporadically. This indicates that stochastic processes are the primary drivers of local influenza A virus evolution, with limited instances of selection‐driven adaptation. The estimated overall mean dN/dS ratio was in line with the findings of other regional studies [29, 31]. The BSP in our study showed low genetic diversity within the Bhutanese strains. Reports have shown that high genetic diversity of the influenza viruses were observed in the global cities with the higher density of migration and subtropical monsoon climate [32]. The low relative genetic diversity in Bhutan could be due to the smaller sample size, lower population and limited accessibility into the country. The analysis also revealed two distinct surges in relative genetic diversity, which coincide with previously documented epidemic peaks and reflecting the semiannual pattern of influenza seasonality in Bhutan [3, 33, 34]. This correlation between *R*_*e*_ and seasonal influenza activity suggests a potential influence of seasonality on the genetic diversity of circulating strains in Bhutan.

The intralineage reassortment analysis showed a single reassortment event between H3 and N2, with a mean rate of 0.045 events per lineage per year (95% HPD: 0.00075–0.174). This rate is significantly lower than the global estimate of 0.35–0.65 events per lineage per year [19]. Similar observations were reported with the Ugandan A(H3N2) strains, where reassortment rates were also lower than global estimates [35]. This may be due to negative selection pressures acting on the resulting reassortant strains, reducing their frequency and likelihood of detection in the population [35, 36, 37]. Our phylogeographic analysis revealed the transmission of A(H3N2) Subclades 2a.3a and 2a.3b from North America to Bhutan in 2022, with simultaneous spread to other Asian and Southeast Asian countries. In 2023, however, Subclade 2a.3a.1 was introduced into Bhutan from Australia. Our results align with global trends, highlighting that A(H3N2) epidemics are influenced by regional transmission hubs, including East‐Southeast Asia and North America. This supports the consensus that the global persistence of A(H3N2) is driven by temporally migrating metapopulations, with new strains emerging across different geographic regions and the location of source populations shifting over time [38, 39].

A major limitation of this study is the availability of genomic sequence data for influenza A(H3N2) viruses in Bhutan, which was restricted to 2022 and 2023. Although the GISAID database contains influenza A(H3N2) sequences from Bhutan dating back to 2009, these earlier sequences were excluded due to their small number and uneven sampling, which would have introduced significant bias into the analysis. Future studies should be done including more comprehensive temporal sampling to enhance the robustness and detail of phylogenetic and evolutionary analyses.

## 5 Conclusion

In conclusion, our study revealed that Bhutanese A(H3N2) strains belong to Clade 3C.2a1b.2a.2. We observed an increase in virus population size in August 2022 and March 2023, aligning with Bhutan's semiannual influenza seasonality. The antigenic profile of the Bhutanese strains remained relatively stable, with the strains primarily under purifying selection and minimal adaptive changes. Our analysis also suggests that the Bhutanese A(H3N2) strains likely originated from the United States in 2022 and from Australia in 2023. This study provides valuable insights into the genetic and evolutionary dynamics of influenza A(H3N2) in Bhutan. Continued monitoring is crucial to deepen our understanding of influenza evolution and refine public health strategies for managing emerging strains effectively.

## Author Contributions

**Tshering Dorji:** conceptualization, methodology, software, investigation, validation, formal analysis, data curation, writing – review and editing, writing – original draft, visualization. **Kunzang Dorji:** conceptualization, writing – review and editing, data curation, methodology, validation, investigation. **Sonam Gyeltshen:** conceptualization, methodology, data curation, writing – review and editing, validation, investigation.

## Conflicts of Interest

The authors declare no conflicts of interest.

## Supporting information


**Figure S1** Temporal Analysis of A(H3N2) HA Gene Divergence.
**Figure S2** Estimates of MCC networks and reassortment rates in Bhutanese A(H3N2).

**Figure S3** Animated GIF showing the phylogeographic spread of A(H3N2) strains into Bhutan. The viral strains circulating in Bhutan in 2022 were introduced from the United States, with additional strains from Australia introduced in 2023. The spatial reconstruction was conducted using SPREAD v1.0.7, where the connections between countries represent branches in the MCC tree indicating location transitions. The map is visualized using satellite imagery from Google Earth.

**Table S1** EPI_SET ID and the acknowledgement for each influenza A(H3N2) sequences used in this study.

**Table S2** Bayes factor and Posterior Probability of migration events of influenza viruses A(H3N2) between Bhutan and other countries in 2022 and 2023.

## Data Availability

The influenza A(H3N2) sequences included in this study are retrieved from EpiFlu™ Database of Global Initiative on Sharing All Influenza Data (GISAID, at https://gisaid.org/). The EPI SET ID of all the sequences is provided in Table S1.
