# Supplementary material for: Evolution of Influenza A(H3N2) Viruses in Bhutan for Two Consecutive Years, 2022 and 2023
Source: Influenza Other Respir Viruses. 2024 Oct 23;18(10):e70028. doi: 10.1111/irv.70028 (PMC11498999; doi:10.1111/irv.70028)

Supplementary Figure S1. Temporal Analysis of A(H3N2) HA Gene Divergence

The figure illustrates the regression of root-to-tip genetic distance against the sampling date for the dataset 1 (A(H3N2)-HA gene). A strong positive correlation is evident, with a correlation coefficient (r) of 0.97 and a coefficient of determination R squared of 0.95, indicating the dataset's suitability for phylogenetic molecular clock analysis in BEAST

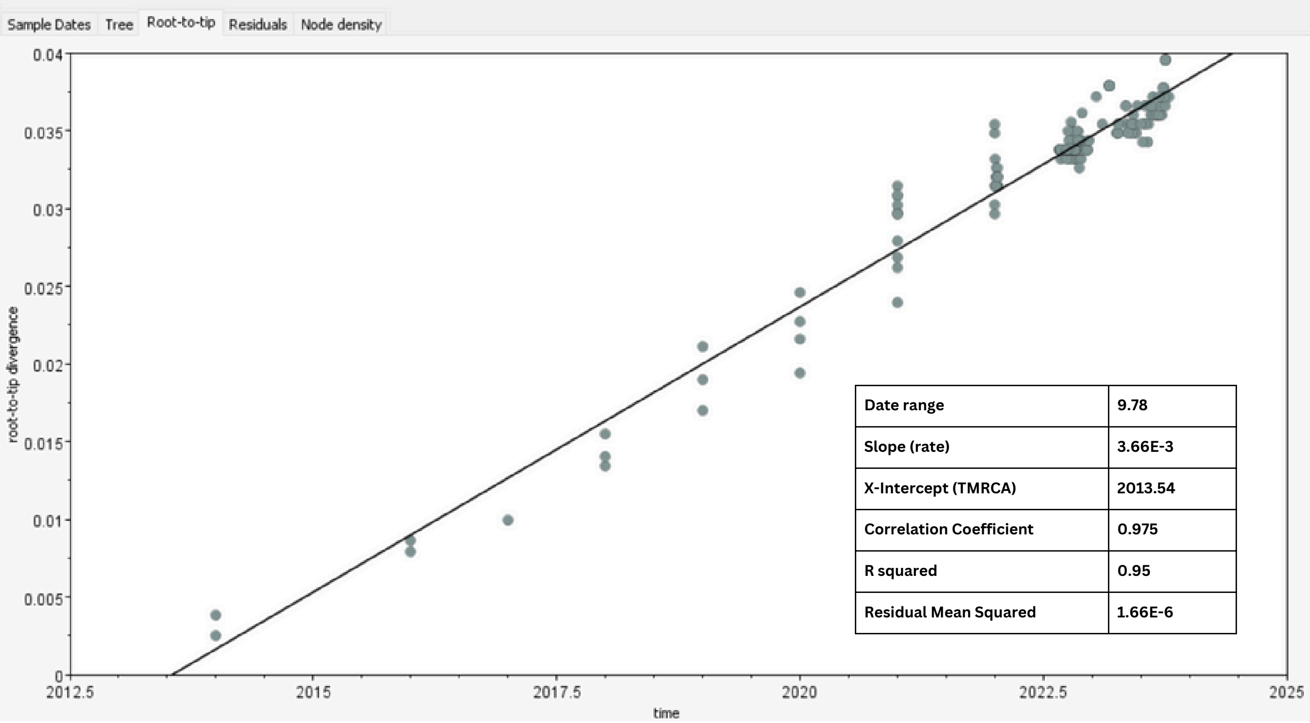

Supplementary Figure S2. Estimates of MCC networks and reassortment rates in Bhutanese A(H3N2) viruses

Estimates of MCC reassortment network between HA and NA genes of Bhutanese influenza A(H3N2) viruses sampled in 2022 and 2023. Icytree visualizes the network as a base tree connected by dotted branches, indicating a reassortment event. The node bars indicate the 95% HPD.

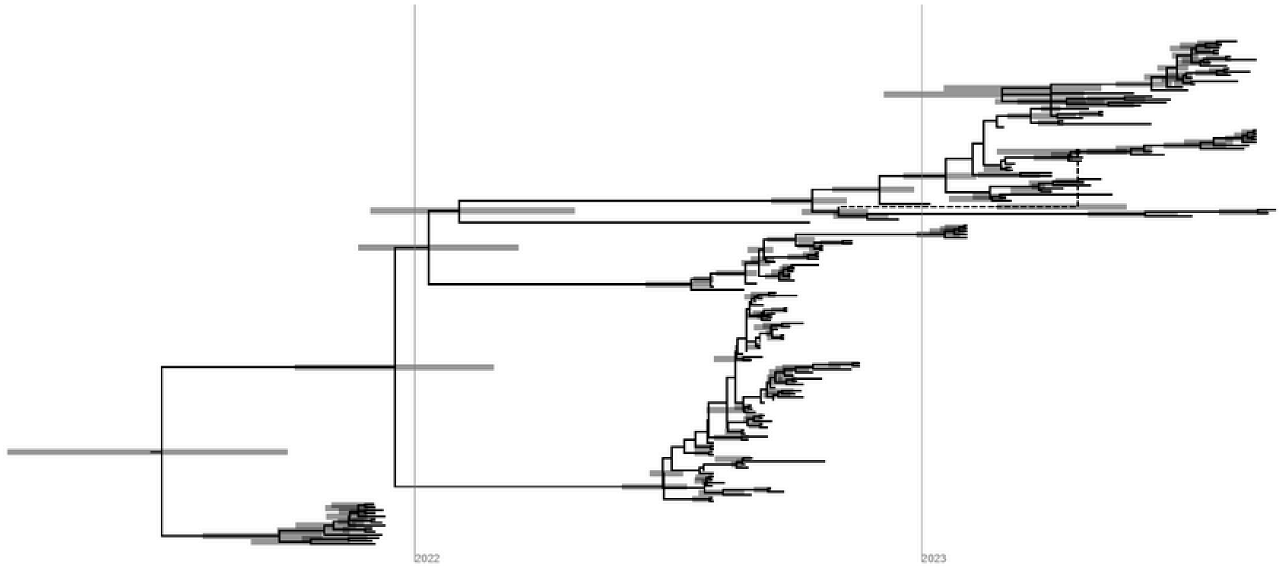

Supplement: Supplementary file 1 — Figure S1 Temporal Analysis of A(H3N2) HA Gene Divergence. Figure S2 Estimates of MCC networks and reassortment rates in Bhutanese A(H3N2). [file IRV-18-e70028-s003.pdf]
