# Supplementary material for: Evolution of Influenza A(H3N2) Viruses in Bhutan for Two Consecutive Years, 2022 and 2023
Source: Influenza Other Respir Viruses. 2024 Oct 23;18(10):e70028. doi: 10.1111/irv.70028 (PMC11498999; doi:10.1111/irv.70028)

**Supplementary Figure S3:** Animated GIF showing the phylogeographic spread of A(H3N2) strains into Bhutan. The viral strains circulating in Bhutan in 2022 were introduced from the United States, with additional strains from Australia introduced in 2023. The spatial reconstruction was conducted using SPREAD v1.0.7, where the connections between countries represent branches in the MCC tree indicating location transitions. The map is visualized using satellite imagery from Google Earth.


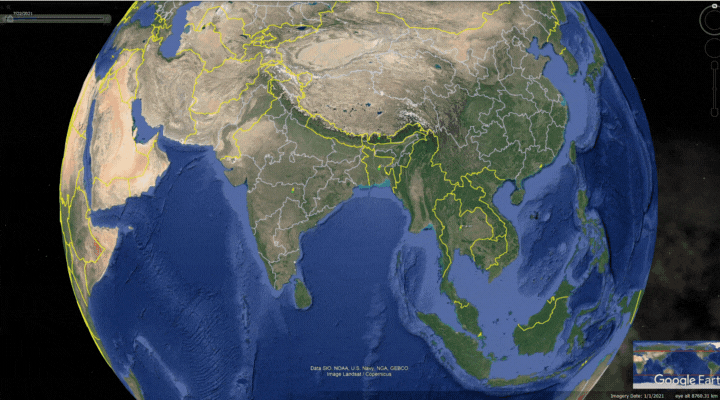

Supplement: Supplementary file 2 — Figure S3 Animated GIF showing the phylogeographic spread of A(H3N2) strains into Bhutan. The viral strains circulating in Bhutan in 2022 were introduced from the United States, with additional strains from Australia introduced in 2023. The spatial reconstruction was conducted using SPREAD v1.0.7, where the connections between countries represent branches in the MCC tree indicating location transitions. The map is visualized using satellite imagery from Google Earth. [file IRV-18-e70028-s004.docx]
