# Supplementary material for: Evolution of Influenza A(H3N2) Viruses in Bhutan for Two Consecutive Years, 2022 and 2023
Source: Influenza Other Respir Viruses. 2024 Oct 23;18(10):e70028. doi: 10.1111/irv.70028 (PMC11498999; doi:10.1111/irv.70028)
Supplement: Supplementary file 4 — Table S2 Bayes factor and Posterior Probability of migration events of influenza viruses A(H3N2) between Bhutan and other countries in 2022 and 2023. [file IRV-18-e70028-s001.docx]

**Table S2.** Bayes factor and Posterior Probability of migration events of influenza viruses A(H3N2) between Bhutan and other countries in 2022 and 2023

| **Posterior Probabilities** | **Bayes Factor** | **Location** |
| --- | --- | --- |
| 1.00 | 648.67 | between Bhutan (long: 90.4336; lat: 27.514162) and Singapore (long: 103.85007; lat: 1.28967) |
| 0.99 | 477.92 | between Bangladesh (long: 90.35633; lat: 23.684994) and United_Kingdom (long: -3.435973; lat: 55.37805) |
| 0.99 | 393.13 | between Bangladesh (long: 90.35633; lat: 23.684994) and HongKong_China (long: 114.167; lat: 22.3) |
| 0.99 | 338.17 | between Bhutan (long: 90.4336; lat: 27.514162) and Thailand (long: 100.99254; lat: 15.870032) |
| 0.99 | 332.25 | between HongKong_China (long: 114.167; lat: 22.3) and Singapore (long: 103.85007; lat: 1.28967) |
| 0.99 | 303.17 | between Australia (long: 133.77513; lat: -25.274399) and Thailand (long: 100.99254; lat: 15.870032) |
| 0.99 | 279.52 | between Singapore (long: 103.85007; lat: 1.28967) and Thailand (long: 100.99254; lat: 15.870032) |
| 0.99 | 244.67 | between India (long: 78.96288; lat: 20.593683) and Singapore (long: 103.85007; lat: 1.28967) |
| 0.99 | 235.33 | between HongKong_China (long: 114.167; lat: 22.3) and United_States (long: -95.71289; lat: 37.09024) |
| 0.98 | 200.39 | between Bhutan (long: 90.4336; lat: 27.514162) and United_Kingdom (long: -3.435973; lat: 55.37805) |
| 0.98 | 188.72 | between India (long: 78.96288; lat: 20.593683) and Thailand (long: 100.99254; lat: 15.870032) |
| 0.98 | 163.32 | between Thailand (long: 100.99254; lat: 15.870032) and United_Kingdom (long: -3.435973; lat: 55.37805) |
| 0.98 | 160.13 | between Australia (long: 133.77513; lat: -25.274399) and Bangladesh (long: 90.35633; lat: 23.684994) |
| 0.98 | 157.86 | between Bangladesh (long: 90.35633; lat: 23.684994) and Thailand (long: 100.99254; lat: 15.870032) |
| 0.97 | 119.34 | between HongKong_China (long: 114.167; lat: 22.3) and Thailand (long: 100.99254; lat: 15.870032) |
| 0.97 | 101.64 | between United_Kingdom (long: -3.435973; lat: 55.37805) and United_States (long: -95.71289; lat: 37.09024) |
| 0.97 | 98.74 | between HongKong_China (long: 114.167; lat: 22.3) and India (long: 78.96288; lat: 20.593683) |
| 0.97 | 95.47 | between Bangladesh (long: 90.35633; lat: 23.684994) and India (long: 78.96288; lat: 20.593683) |
| 0.97 | 95.24 | between Australia (long: 133.77513; lat: -25.274399) and Singapore (long: 103.85007; lat: 1.28967) |
| 0.97 | 87.89 | between Bhutan (long: 90.4336; lat: 27.514162) and HongKong_China (long: 114.167; lat: 22.3) |
| 0.96 | 77.66 | between HongKong_China (long: 114.167; lat: 22.3) and United_Kingdom (long: -3.435973; lat: 55.37805) |
| 0.96 | 66.68 | between Thailand (long: 100.99254; lat: 15.870032) and United_States (long: -95.71289; lat: 37.09024) |
| 0.94 | 51.84 | between India (long: 78.96288; lat: 20.593683) and United_Kingdom (long: -3.435973; lat: 55.37805) |
| 0.94 | 46.82 | between Bangladesh (long: 90.35633; lat: 23.684994) and United_States (long: -95.71289; lat: 37.09024) |
| 0.93 | 40.41 | between Singapore (long: 103.85007; lat: 1.28967) and United_States (long: -95.71289; lat: 37.09024) |
| 0.92 | 38.49 | between Bhutan (long: 90.4336; lat: 27.514162) and India (long: 78.96288; lat: 20.593683) |
| 0.91 | 30.94 | between Bangladesh (long: 90.35633; lat: 23.684994) and Bhutan (long: 90.4336; lat: 27.514162) |
| 0.83 | 15.31 | between Bhutan (long: 90.4336; lat: 27.514162) and United_States (long: -95.71289; lat: 37.09024) |
| 0.82 | 14.23 | between Australia (long: 133.77513; lat: -25.274399) and United_Kingdom (long: -3.435973; lat: 55.37805) |
| 0.69 | 6.89 | between Bangladesh (long: 90.35633; lat: 23.684994) and Singapore (long: 103.85007; lat: 1.28967) |
| 0.65 | 5.94 | between Australia (long: 133.77513; lat: -25.274399) and Bhutan (long: 90.4336; lat: 27.514162) |
